# Genome-Wide Identification, Expression and Functional Analysis Reveal the Involvement of FCS-Like Zinc Finger Gene Family in Submergence Response in Rice

**DOI:** 10.1186/s12284-021-00519-3

**Published:** 2021-08-21

**Authors:** Yamei Ma, Junliang Zhao, Hua Fu, Tifeng Yang, Jingfang Dong, Wu Yang, Luo Chen, Lian Zhou, Jian Wang, Bin Liu, Shaohong Zhang, David Edwards

**Affiliations:** 1 Rice Research Institute, Guangdong Academy of Agricultural Sciences, Guangzhou, 510640 China; 2 Guangdong Key Laboratory of New Technology in Rice Breeding, Guangzhou, 510640 China; 3 School of Biological Sciences and Institute of Agriculture, The University of Western Australia, Perth, WA Australia

**Keywords:** FLZ gene family, *OsFLZ18*, *SnRK1A*, Submergence, Rice

## Abstract

**Background:**

Direct seeding is an efficient rice cultivation practice. However, its application is often limited due to O_2_ deficiency following submergence, leading to poor seed germination, seedling establishment, and consequently yield loss. Identification of genes associated with tolerance to submergence and understanding their regulatory mechanisms is the fundamental way to address this problem. Unfortunately, the molecular mechanism of rice response to submergence stress is still not well understood.

**Results:**

Here, we have performed a genome-wide identification of FCS-like zinc finger (FLZ) proteins and assessed their involvement in submergence response in rice. We identified 29 FLZ genes in rice, and the expression analysis revealed that several genes actively responded to submergence stress. Eight OsFLZ proteins interact with SnRK1A. As a case study, we demonstrated that OsFLZ18 interacted with SnRK1A and inhibited the transcriptional activation activity of SnRK1A in modulating the expression of its target gene *αAmy3*, a positive regulator in rice flooding tolerance. In line with this, *OsFLZ18*-overexpression lines displayed retarded early seedling growth and shorter coleoptile following submergence.

**Conclusions:**

These data provide the most comprehensive information of *OsFLZ* genes in rice, and highlight their roles in rice submergence response.

**Supplementary Information:**

The online version contains supplementary material available at 10.1186/s12284-021-00519-3.

## Background

Rice (*Oryza sativa* L.) is a staple food for over 50% of the world population. To meet the demand for the increasing population, 114 million tons of additional milled rice must be produced by 2035, which is equivalent to an overall increase of 26% in the next 25 years (Kumar and Ladha 2011). However, rice production is frequently affected by different stresses. Low oxygen stress caused by flooding or submergence is one of the major abiotic stresses that affect the development and growth of rice (Miro and Ismail 2013). The rapid decline in oxygen (O_2_) diffusion rate (10,000-fold less) during flooding is accompanied by a reduction in cellular O_2_ level and an energy crisis, which are particularly severe when photosynthesis is limited or absent (Jackson and Ram 2003; Bailey-Serres and Voesenek 2008; Licausi and Perata 2009). Mature plants for most rice varieties die within 14 days of complete submergence (Xu et al. 2006). Additionally, low oxygen stress caused by submergence is one of the major factors limiting the application of rice direct seeding, which is a newly emerging agricultural practice and increasingly accepted by the farmers due to reduced need for water and labor, and efficient utilization of resources compared with the traditional transplanting practice (Kumar and Ladha 2011; Liu et al. 2015). However, the submerged seeds in rice direct seeding are subject to O_2_ deficiency, which leads to poor germination rate and seedling establishment, and consequently yield loss.

Identification of the genes associated with tolerance to submergence stress and understanding their regulatory mechanisms is a fundamental way to address this problem. Over the past 2 decades, significant progress has been made in understanding the molecular and physiological mechanisms of rice in response to submergence stress. The most exciting event is the discovery of *SUB1*, a robust quantitative trait locus (QTL) for submergence tolerance that encodes a variable cluster of three ERF genes: *Sub1A*, *Sub1B* and *Sub1C* (Fukao et al. 2006; Xu et al. 2006). Studies showed that Sub1A enabled mature plants to tolerate up to 14 days of complete submergence by stimulating the expression of a gene encoding alcohol dehydrogenase (Adh), an enzyme necessary for fermentative metabolism; and repressing GA-mediated induction of genes encoding α-amylase and expansin, which are respectively involved in starch degradation and cell elongation in leaves, thereby preserving energy until floodwaters recede (Fukao et al. 2006; Fukao and Bailey-Serres 2008). These results well explain the molecular and physiological mechanisms of the mature rice plant to cope with submergence stress. However, the seed germination and coleoptile elongation of some rice varieties without *Sub1A* proceed satisfactorily under hypoxic or anoxic conditions (Magneschi and Perata 2009), indicating that these rice cultivars might use a Sub1A-independent mechanism to cope with low-O_2_ stress during germination and seedling establishment.

In the past decade, progress has been made in understanding the regulatory mechanism of rice seed germination and seedling growth under hypoxic condition. Studies suggest that SUCROSE NONFERMENTING 1-RELATED PROTEIN KINASE 1A (SnRK1A) acts as a central regulator of seed germination and seedling growth in rice by controlling the transcriptional activation of *MYELOBLASTOSIS SUCROSE 1*(*MYBS1*) and *αAmy3* (Lu et al. 2007; Lee et al. 2009, 2014). Submergence-induced SnRK1A-INTERACTING NEGATIVE REGULATOR 1/2 (SKIN1/2) represses SnRK1A-dependent sugar and nutrient starvation signaling pathway to negatively regulate rice submergence tolerance (Lin et al. 2014). Conversely, trehalose-6-phosphate phosphatase (TPP7) derepresses trehalose-6-phosphate (T6P)-mediated suppression of SnRK1A activity, thus enhancing the tolerance of rice to submergence (Kretzschmar et al., 2015). Furthermore, CIPK15, a CALCINEURIN B-LIKE (CBL) PROTEIN INTERACTING PROTEIN KINASE, acts as the main upstream positive regulator of SnRK1A in rice, and directly interacts with SnRK1A to integrate O_2_-deficiency signaling with SnRK1A-MYBS1-mediated sugar signaling to monitor sugar and energy production, allowing rice to grow better under submergence conditions (Lee et al. 2009, 2014). However, these studies only provide a framework and much remains to be learned about the biochemical and molecular basis of rice germination and seedling growth under submergence stress.

FCS-like zinc finger (FLZ) proteins are plant-specific regulatory proteins containing a common FLZ domain or DUF581 (Domain of Unknown Function 581). Recently, several reports demonstrated that FLZ proteins might function as the scaffolding proteins of SnRK1 by interacting with SnRK1 kinase subunits (Arabidopsis Interactome Mapping Consortium 2011; Nietzsche et al. 2014, 2016; Jamsheer et al. 2018a, b). In *Arabidopsis*, AtFLZ6/10 interacts with SnRK1α to repress SnRK1 signaling through inhibiting SnRK1α protein accumulation. In line with this, the *flz6* and *flz10* knockdown mutants phenocopied the SnRK1α overexpression plants with higher SnRK1α protein levels and retarded growth (Jamsheer et al. 2018a). Gene expression analysis showed that *AtFLZ* genes were significantly regulated by sugars, ABA, and environmental cues, including hypoxia and light, which could actively affect the activity of SnRK1 (Nietzsche et al. 2014; Jamsheer and Laxmi 2015; Jamsheer et al. 2018a). Since the central role of SnRK1 in regulating plant response to submergence stress has been well established (Lu et al. 2007; Lee et al. 2009; Cho et al. 2012; Chen et al. 2017), and there are strong interactions between FLZ proteins and SnRK1 in *Arabidopsis* and maize (Arabidopsis Interactome Mapping Consortium 2011; Nietzsche et al. 2014, 2016; Jamsheer et al. 2018a, b; Chen et al. 2021), we reason that the FLZ proteins might also function in submergence stress in rice by interacting with SnRK1A. However, the characterization of FLZ proteins in rice has not yet been reported.

To test our hypothesis, we performed a genome-wide identification analysis of proteins with a conserved FLZ domain in the rice genome, confirmed the interactions between SnRK1A and OsFLZs, investigated OsFLZ subcellular localizations, and assessed the expression patterns of *OsFLZ* genes in different tissues and under submergence. Furthermore, as a case study, we functionally confirmed the role of the submergence responsive FLZ protein OsFLZ18 in regulating early seedling growth through interacting with SnRK1A. This study for the first time provides fundamental information on FLZ proteins in rice and demonstrates their potential roles in submergence stress and other abiotic stresses.

## Results

### Identification and Phylogenetic Analysis of Rice FLZs

To identify candidate FLZ family proteins in rice, the Pfam term DUF581 was used as a query to search the Rice Genome Annotation Project database (RGAP, http://rice.plantbiology.msu.edu/). In total, 29 independent rice OsFLZs were identified. This result was confirmed using the PLAZA v 4.5 database. The 29 FLZs are named OsFLZ1 to OsFLZ29 based on their distribution and relative linear order on rice chromosomes (Additional file 1: Table S1, Additional file 2: Fig. S1). Detail information about each OsFLZ, including gene name, locus, chromosome location (start site and end site), nucleotide length, predicted protein molecular weight, isoelectric point, and protein subcellular localization is given in Additional file 1: Table S1. The coding region of the *OsFLZ* genes varied from 309 (*OsFLZ21*) to 1167 (*OsFLZ1*) base pairs (bps), encoding 103 to 389 amino acids, with molecular weights from 10.76 to 39.42 kilo Dalton (kDa) and isoelectric points from 4.07 to 11.56 (Additional file 1: Table S1). Subcellular localization was predicted using WoLF pSORT (http://www.genscript.com/psort/wolf_psort.html), which showed that OsFLZs proteins were potentially localized in the nucleus, cytoplasm, chloroplast, mitochondria, extracellular and endoplasmic reticulum (Additional file 1: Table S1).

**Fig. 1:**
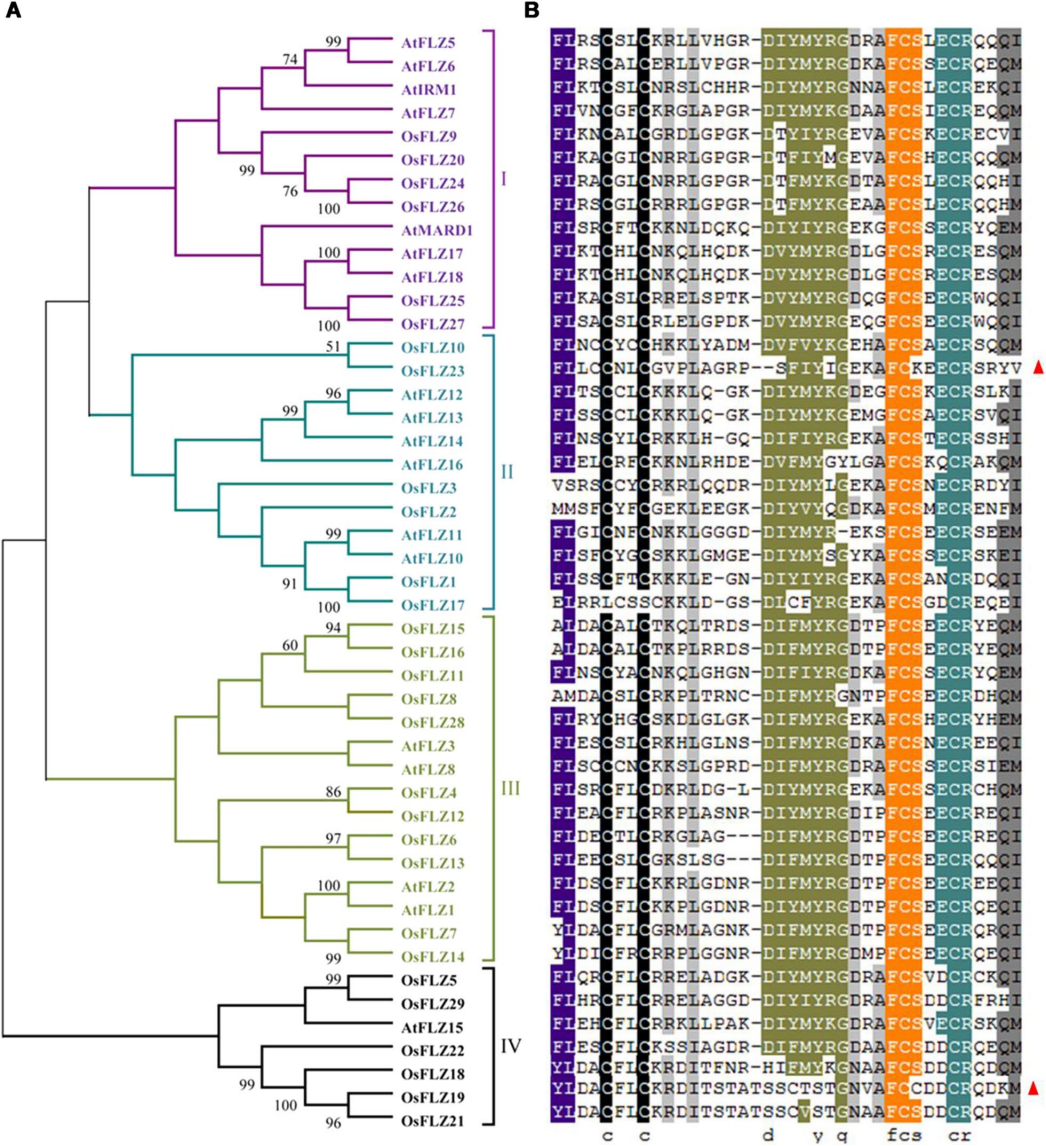
Phylogenetic and conserved domain analysis of FLZ proteins from rice and Arabidopsis. **a** Phylogenetic relationships of FLZ proteins between rice and Arabidopsis. The phylogenetic tree was constructed based on the multiple sequence alignments of FLZ proteins and generated with MEGA 7.0 software using the Neighbor-Joining method. Bootstrap values from 1000 replicates are indicated at each branch. **b** Alignment of the conserved FLZ structural domains. The conserved FLZ amino acid signature is highlighted in different color, and gaps are marked with dashes. Triangles indicate that OsFLZ19 and OsFLZ23 share a single amino acid difference. The structure maps were drawn with Genedoc

To explore the phylogenetic relationship among FLZ proteins, full-length FLZ protein sequences from rice and *Arabidopsis* were used to construct an unrooted phylogenetic tree via Neighbor Joining method (Fig. 1a). These proteins grouped in four clades based on their sequence identity. Clade III is the largest group with 11 OsFLZs, and each of the other three clades contains 6 members (Fig. 1a). A multiple sequence alignment of the core FLZ domain, representing around 40 amino acids from FLZ proteins from rice and *Arabidopsis*, is illustrated in Fig. 1b. Except for OsFLZ19 and OsFLZ23, all other 27 OsFLZ proteins share highly conserved sequence identity (CX_2_CX_17–19_FCSX_2_C), while OsFLZ19 and OsFLZ23 share a single amino acid difference (Fig. 1b). Furthermore, many other residues are equally conserved in the FLZ domain region between rice and *Arabidopsis*.

### The Expression Pattern of *OsFLZ* Genes in Different Tissues

**Fig. 2:**
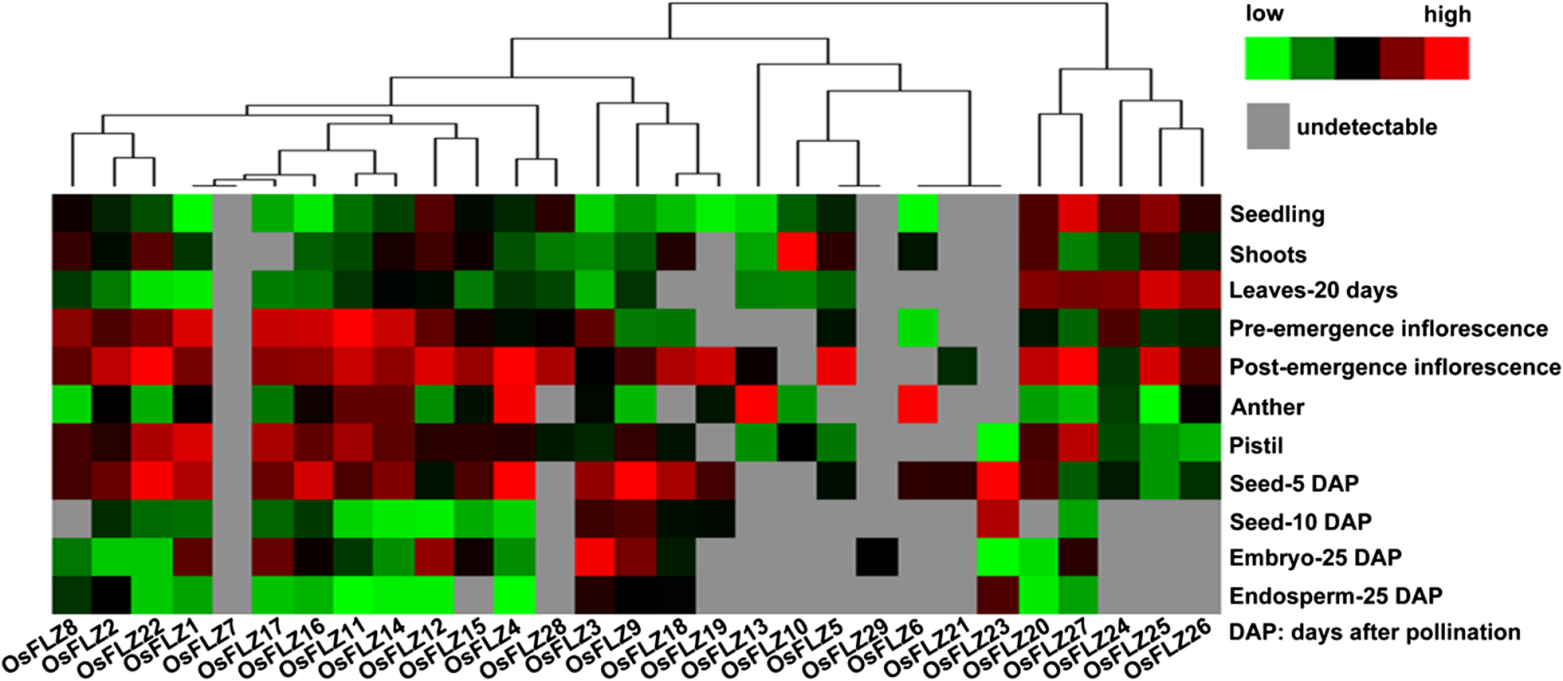
Heatmap of the gene expression of *OsFLZs* in 11 different tissues. The color scale represents relative expression level from low (green) to high values (red). The raw data of gene expression were downloaded from Rice Genome Annotation Project (http://rice.plantbiology.msu.edu/)

To understand the potential function of *OsFLZ* genes in regulating rice growth and development, the spatial expression patterns of 29 *OsFLZ* genes were analyzed. We downloaded the RNA-Seq data for 11 different tissues/organs of *japonica* rice cv. *Nipponbare* from the Rice Genome Annotation Project (http://rice.plantbiology.msu.edu/) to generate a heatmap using Cluster 3 software. As shown in Fig. 2, most *OsFLZ* genes were ubiquitously but differentially expressed in various tissues and *OsFLZ1*, *OsFLZ2*, *OsFLZ3*, *OsFLZ4*, *OsFLZ9*, *OsFLZ11*, *OsFLZ12*, *OsFLZ14*, *OsFLZ16*, *OsFLZ22* and *OsFLZ27* were constitutively expressed in all 11 tested samples. The highest expression levels of *OsFLZ6* and *OsFLZ13* were observed in anther, while *OsFLZ5* and *OsFLZ10* exhibited the highest expression in post-emergence inflorescence and shoots, respectively. *OsFLZ29* was only found to be expressed in the embryo 25 days after pollination (Embryo-25 DAP) at a very low level, and no expression was detected for *OsFLZ7* in any of the tissues examined. The diverse expression patterns of *OsFLZ* genes indicated that they might exert different functions in rice growth and development.

### Subcellular Localization of OsFLZs-GFP Fusions

**Fig. 3:**
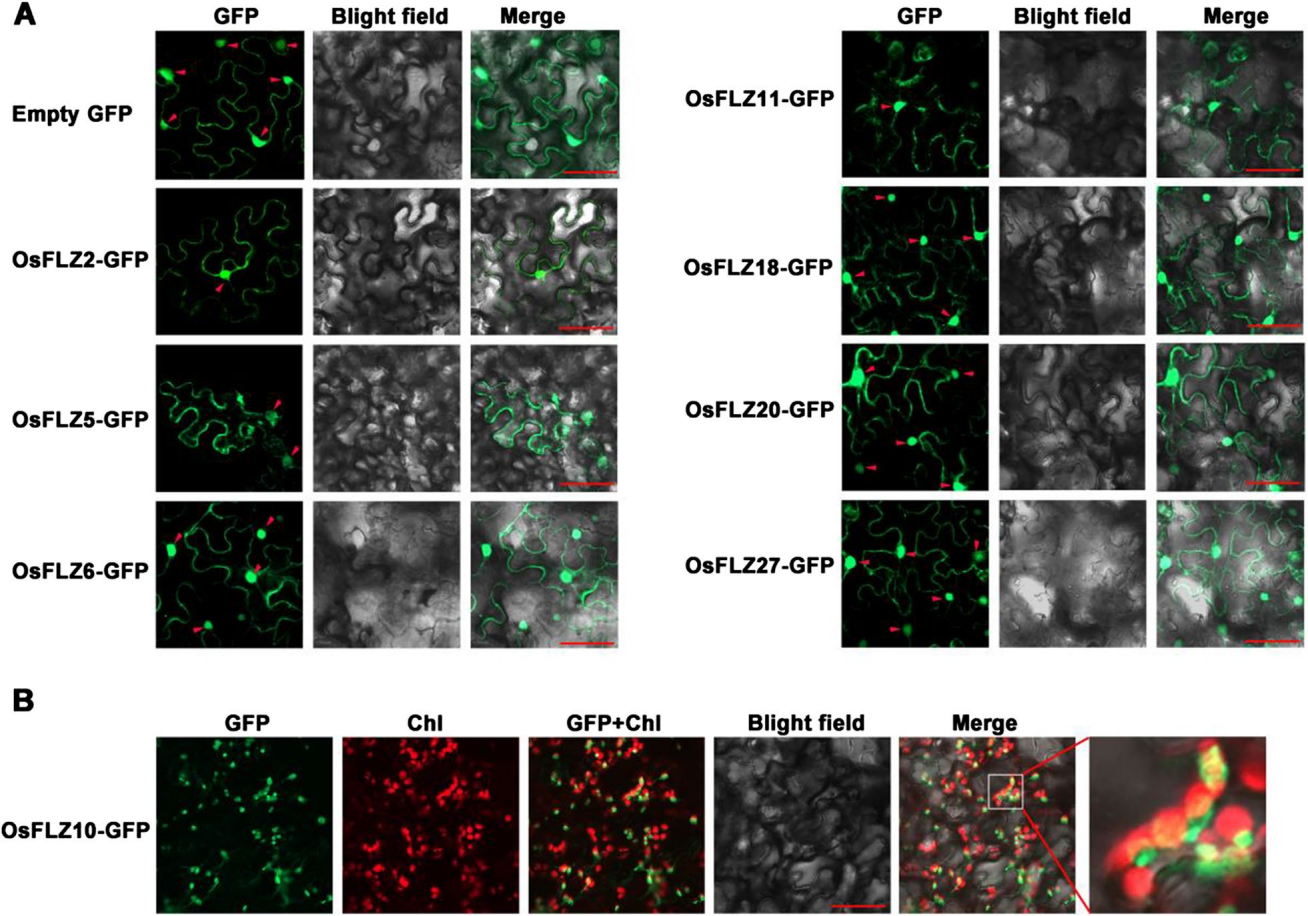
Subcellular localization of OsFLZ-GFP proteins. **a** OsFLZ-GFP fusion constructs or empty GFP were expressed transiently in tobacco epidermal cells, respectively. The signals of GFP and chlorophyll autofluorescence (Chl) were captured with a confocal laser scanning microscopy and shown in green and red, respectively. Red arrows point to the cell nucleus. **b** OsFLZ10-GFP was partially co-localized with chloroplast. Scale bars: 50 μm

Subcellular localization of OsFLZs predicted by WoLF PSORT suggested that they were localized in diverse subcellular compartments (Additional file 1: Table S1), indicating multifunctional roles in different cellular processes. To confirm the subcellular localization of a subset of OsFLZ proteins, eight representative OsFLZs from four different clades, OsFLZ20 and OsFLZ27 from clade I, OsFLZ10 and OsFLZ2 from clade II, OsFLZ11 and OsFLZ6 from clade III, OsFLZ5 and OsFLZ18 from clade IV, were chosen to fuse in-frame with green fluorescence protein (GFP) driven by a maize ubiquitin promoter, and the resultant chimeric proteins were transiently expressed in 6-week-old tobacco (*Nicotiana benthamiana*) leaf epidermal cells. Confocal scanning microscopy revealed that the GFP signals of OsFLZ2, OsFLZ5, OsFLZ6, OsFLZ11, OsFLZ18, OsFLZ20 and OsFLZ27 fusions were found both in the nucleus and the cytoplasm, as was the case of empty GFP protein (Fig. 3a). Interestingly, OsFLZ10-GFP was shown to target the granular spots in the cytoplasm, which partially co-localized with chloroplast (Fig. 3b). The results are, in general, consistent with the bioinformatics prediction, in which they mainly localize in nucleus and cytoplasm (Additional file 1: Table S1, Fig. 3).

### The Interactions Between OsFLZs and SnRK1A

**Fig. 4:**
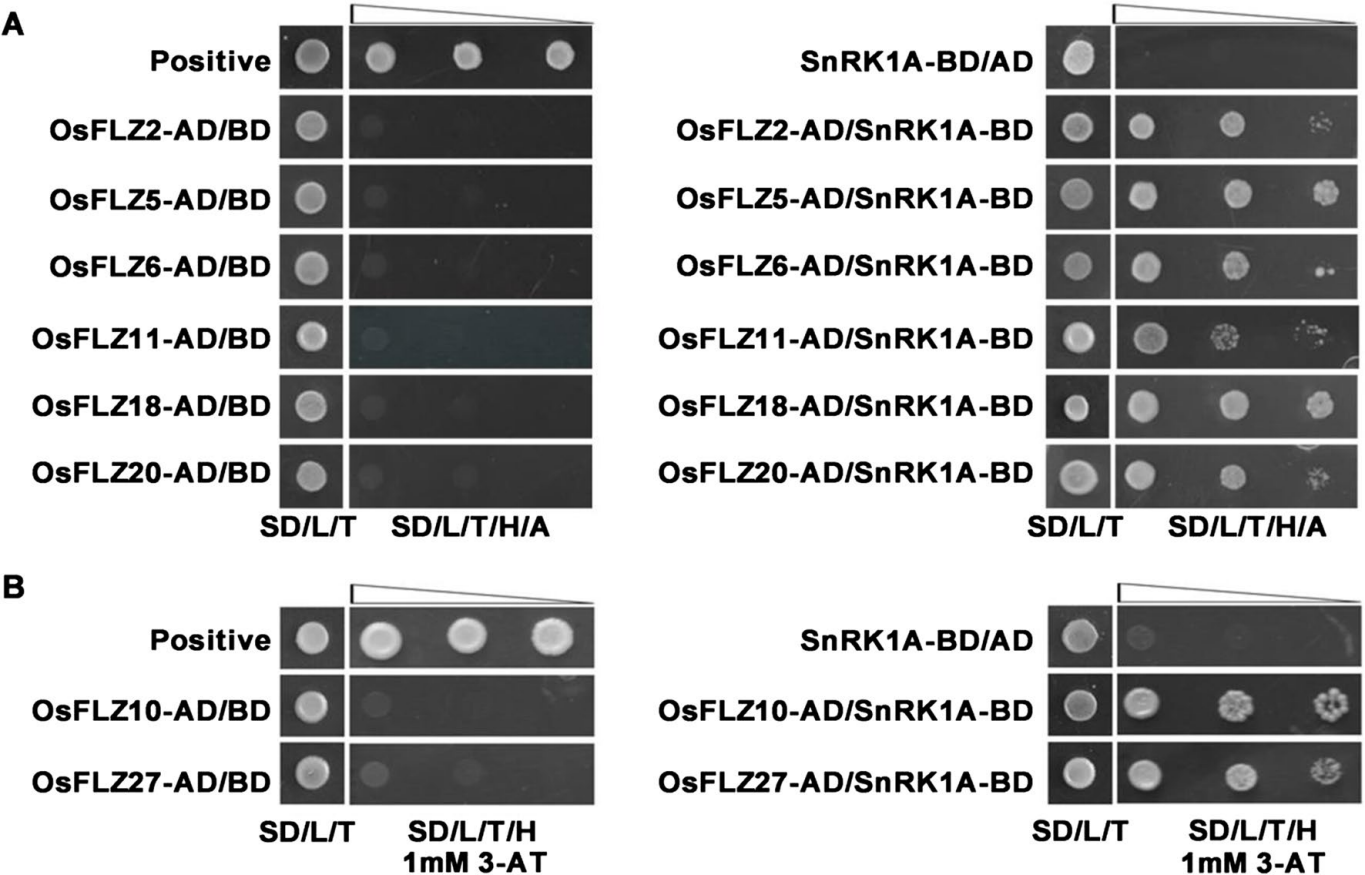
OsFLZs interact with SnRK1A in yeast. OsFLZs were fused to the activation domain, and rice SnRK1A was fused to the DNA-binding domain, respectively. Co-transformed yeast clones were serial diluted and then assessed on SD/–Leu–Trp–His-Ade (**a**) or SD/–Leu–Trp–His+ 1 mM 3AT plates (**b**) to test the interaction. The combination of pGBKT7-53 and pGADT7-T was used as a positive control

The essential role of SnRK1A in rice submergence tolerance has been well established (Lu et al. 2007; Lee et al. 2009; Cho et al. 2012). Previous studies showed that FLZs could interact with the subunits of the SnRK1 kinase complex in *Arabidopsis* and maize (Arabidopsis Interactome Mapping Consortium 2011; Nietzsche et al. 2014, 2016; Jamsheer et al. 2018a, b; Chen et al. 2021). To see whether OsFLZs can also interact with SnRK1A to regulate rice response to submergence stress, we selected the above eight representative *OsFLZ* genes to perform a yeast two-hybrid assay (Fig. 4). The full-length OsFLZs were fused to the GAL4 activation domain (OsFLZ-AD), and SnRK1A was fused to the DNA binding domain (SnRK1A-BD) vectors, respectively. Six out of the 8 selected OsFLZs-AD (OsFLZ2, OsFLZ5, OsFLZ6, OsFLZ11, OsFLZ18 and OsFLZ20) strongly interacted with SnRK1A-BD, but not with empty BD vector, as judged by the growth of co-transfected yeast cells on SD–Trp/–Leu/–His/–Ade selective medium plates (Fig. 4a). The other two OsFLZ proteins (OsFLZ10 and OsFLZ27) moderately interacted with SnRK1A, as indicated by the growth of co-transfected yeast cells on SD–Trp/–Leu/–His medium containing 1 mM 3-AT, which was comparable to that of the positive control (Fig. 4b). These results suggest that OsFLZs universally interact with SnRK1A, and these interactions might affect rice tolerance to submergence stress.

### The Response of *OsFLZ* Genes Toward Submergence Stress

**Fig. 5:**
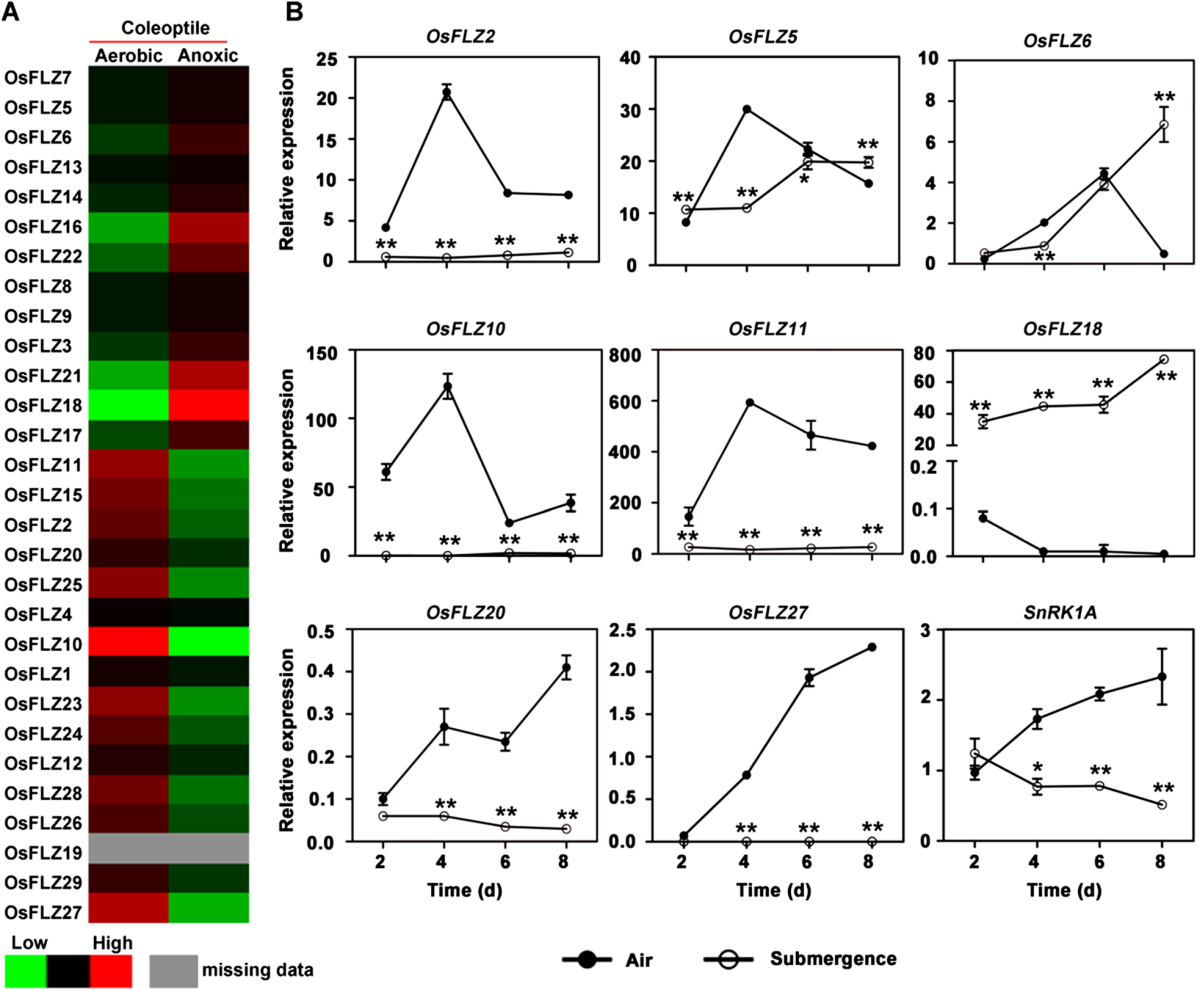
Expression profiles of *OsFLZ* genes under submergence condition. **a** Heatmap based on the microarray data with respect to the relative expression levels of *OsFLZ* genes in coleoptile under aerobic and anoxic conditions (Lasanthi-Kudahettige et al. 2007). **b** Validation of the gene expression of *OsFLZs* over time after submergence treatment by qRT-PCR. The coleoptiles from germinated seeds were then collected over a time course indicated. The transcription levels of rice *OsEF1α* were used as internal controls. *p < 0.05, **p < 0.01, Student’s *t*-test

The above experiments confirmed the strong interactions between OsFLZs and SnRK1A and implied that OsFLZs might function in submergence stress in rice. To confirm this hypothesis, we firstly downloaded and analyzed the transcriptomic data which was derived from *Nipponbare* with respect to aerobic and anoxic germination (Lasanthi-Kudahettige et al. 2007). We found that 10 *OsFLZ* genes were significantly down-regulated, but 5 *OsFLZ* genes were significantly up-regulated, by at least twofold, under anoxia conditions (Fig. 5a), in which *OsFLZ18* exhibited the most significant up-regulation. To confirm the responses of *OsFLZ* genes towards submergence stress, quantitative real-time PCR (qRT-PCR) was used to monitor the transcript abundance of the eight representative *OsFLZ* and *SnRK1A* genes in aerobic or hypoxic coleoptiles of *Nipponbare*. The tested seeds were divided into two groups. One group was submerged in 10 cm water and the other group was kept in air. The coleoptiles from the two groups were collected for total RNA extraction on the 2nd, 4th, 6th, 8th day after treatment, respectively, and qRT-PCR assays were conducted with these samples. The results confirmed that the eight selected *OsFLZ* genes were all submergence-responsive (Fig. 5b). It is worth noting that the transcription of *OsFLZ18* is very drastically up-regulated, with approximately 400-fold increase on the 2nd day and 800-fold increase on the 8th day, respectively (Fig. 5b). Compared to *OsFLZ* genes, the expression of *SnRK1A* was slightly repressed in the hypoxic coleoptile (Fig. 5b). These results suggest that both *OsFLZ* genes and *SnRK1A* are transcriptionally regulated by submergence treatment.

### Functional Confirmation of OsFLZ18 in Regulating Germination and Early Seedling Growth Under Submergence Stress

**Fig. 6:**
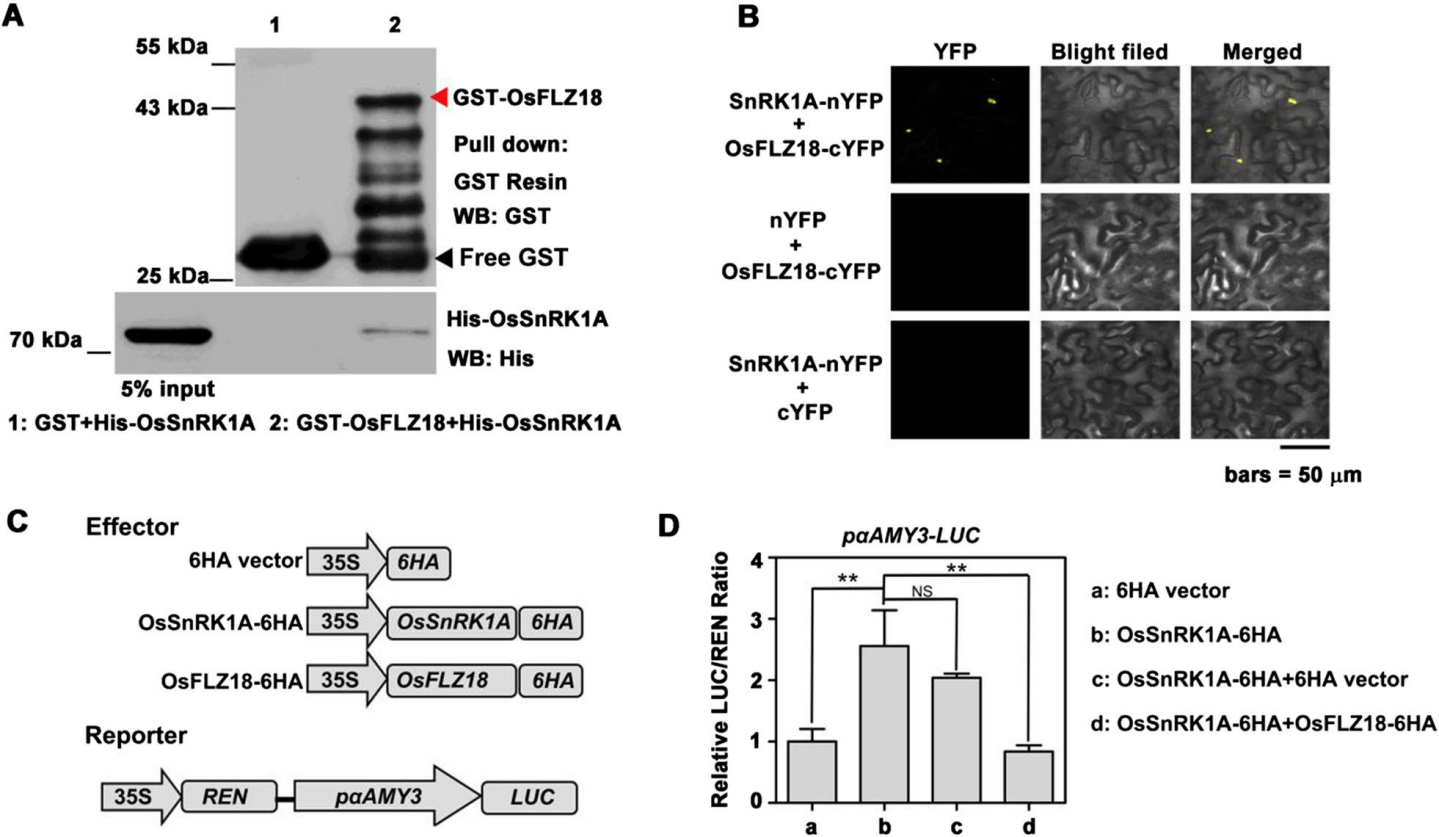
OsFLZ18 physically interacts with and antagonizes the SnRK1A-dependent transcriptional activation to *αAmy3*. **a** Pull-down assay confirmed the interaction between OsFLZ18 and SnRK1A. **b** BiFC assay showed the interaction between OsFLZ18 and SnRK1A in *Nicotiana benthamiana* leaves. Scale bars: 50 μm. **c** Schematic constructs of the reporters and effectors used in the dual-luciferase reporter assay. **d** Dual-luciferase reporter assay showing the inhibition effect of OsFLZ18 on the transcriptional activation of *αAmy3* by SnRK1A. Data are means ± SD from four biological replicates. *NS* no significant, **p < 0.01, Student’s *t* test

*OsFLZ18* is the most strongly responsive *OsFLZ* following submergence stress in this study (Fig. 5b). As a case study, we have confirmed its interaction with SnRK1A and function on submergence stress in rice. As shown in Fig. 6a, His-SnRK1A could bind to GST-OsFLZ18, but not bind to the free GST, indicating that SnRK1A physically interacted with OsFLZ18. This interaction was further confirmed by bimolecular fluorescence complementation (BiFC) assay in tobacco (Fig. 6b). When SnRK1A-nYFP was co-infiltrated with OsFLZ18-cYFP into tobacco leaves, strong YFP signals were observed in the transformed cells. However, no fluorescence was detected in negative controls in which SnRK1A-nYFP was coexpressed with cYFP or OsFLZ18-cYFP was co-expressed with nYFP (Fig. 6b). To assess the potential role of the OsFLZ18-SnRK1A interaction, we employed the widely used dual-luciferase assay to evaluate the effects of OsFLZ18 on the signaling output of SnRK1A, by measuring the transcriptional regulatory activity of SnRK1A to its induced target gene *αAmy3* (Lu et al. 2007). In line with the previous report (Lu et al. 2007), SnRK1A could significantly activate the expression of *αAmy3* (Fig. 6d). Interestingly, addition of OsFLZ18 could effectively antagonizes the SnRK1A-dependent transcriptional activation of *αAmy3* (Fig. 6d), indicating that OsFLZ18 might function as a repressor of SnRK1 signaling.

**Fig. 7:**
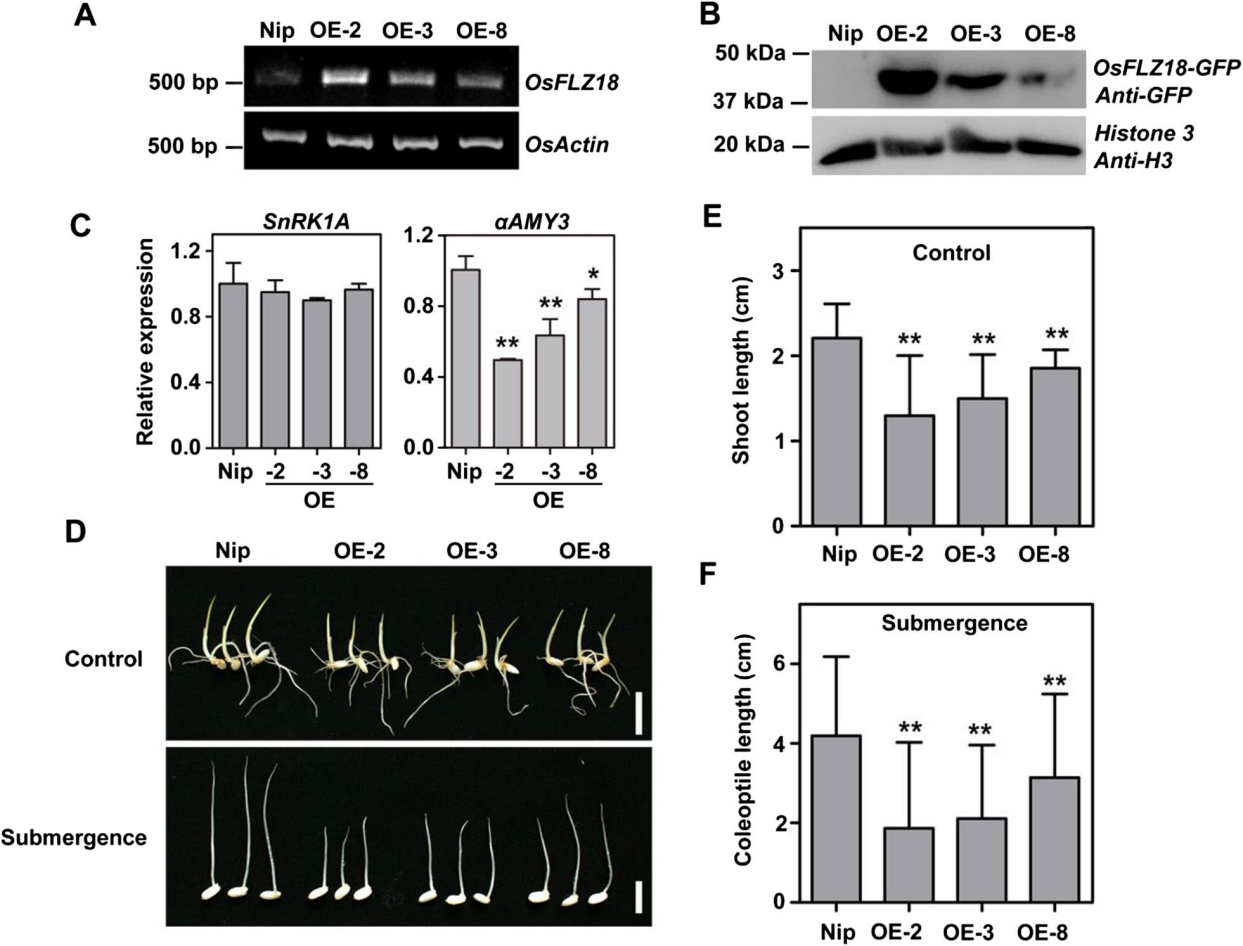
*OsFLZ18* negatively regulates early seedling growth in air and coleoptile elongation under submergence stress. **a** Relative expression of *OsFLZ18* in WT and three OE transgenic lines by qRT-PCR. **b** Protein accumulations of OsFLZ18-GFP in WT and three OE transgenic lines by Western Blotting. Total protein was extracted from seedlings, and antibodies against GFP and Histone 3 were used in this assay. **c** qRT-PCR analysis of the transcriptional levels of *SnRK1A* and *αAmy3* in WT and OE transgenic lines. *p < 0.05, **p < 0.01, Student’s *t*-test. **d** Phenotypes of WT and OE transgenic lines in air and under submerged conditions. Sterile seeds were germinated for 7 days in air and under complete submergence. Bars = 1 cm. **e**, **f** Statistical data for the shoot lengths (**e**) or coleoptile lengths (**f**) of WT and OE transgenic plants shown in (**d**) (n > 30). **p < 0.01, Student’s *t*-test

To further confirm the function of *OsFLZ18* in rice submergence stress, we generated *OsFLZ18* overexpression (OE) transgenic rice plants in *Nipponbare* background. In total, 25 independent *OsFLZ18-GFP* OE lines were obtained, and three homozygous T_3_ lines with high but different gene and protein expression levels were chosen for further phenotypic evaluation (Fig. 7a, b). We also checked the expression levels of *SnRK1A* and *αAmy3* in *Nipponbare* and *OsFLZ18-GFP* OE lines by qRT-PCR assay. The results showed that the expression of *SnRK1A* had no obvious alteration among *Nipponbare* and OE lines, but the expression level of *αAmy3* was significantly decreased in OE lines (Fig. 7c). To investigate whether *OsFLZ18* affects rice growth and development, wild type (WT) and three *OsFLZ18-GFP* OE transgenic seeds were respectively germinated in air or submergence conditions and their phenotypes were compared after growing for 7 days (Fig. 7d). In air, the growth of *OsFLZ18-GFP* OE plants were retarded, with significantly shorter shoots than the wild type plants (Fig. 7d, e). Under the submergence condition, all genotypes failed to generate roots, but the coleoptiles emerged and more elongated in WT than those in *OsFLZ18-GFP* OE lines (Fig. 7d–f). In addition, the shoot lengths in air and coleoptile lengths in submergence were negatively correlated with the gene or protein expression levels. These results together suggest that OsFLZ18 negatively regulate rice growth in both air and submergence conditions.

## Discussion

So far, 757 FLZ proteins have been identified from 41 plant genomes including 29 members in rice through genome-wide identification, and they were plant-specific zinc finger proteins involved in protein–protein interaction (Jamsheer and Laxmi 2014; Jamsheer et al. 2015). Until now, most information on *FLZ* family genes were obtained from studies in *Arabidopsis* and maize (He and Gan 2004; Chen et al. 2013, 2021; Hou et al. 2013; Jamsheer and Laxmi 2014, 2015; Nietzsche et al. 2014, 2016; Jamsheer et al. 2018a, b), and FLZ proteins in other plants have not yet been characterized. In the present study, we have provide the gene and protein attributes, and phylogenetic relationships among the 29 OsFLZ proteins (Additional file 1: Table S1, Fig. 1), as well as gene expression patterns in different tissues (Fig. 2) and their subcellular localizations in rice (Fig. 3). Our results suggest that the core sequence of FLZ proteins between rice and *Arabidopsis* is conserved, though large variations were observed in nucleotide length, protein molecular weight, protein isoelectric point (Additional file 1: Table S1). The FLZ proteins from rice and *Arabidopsis* grouped into four clades based on protein sequence identity (Fig. 1). The proteins were localized in different organelles including the nucleus, cytoplasm, chloroplast and mitochondria (Additional file 1: Table S1, Fig. 3). Additionally, most *OsFLZ* genes were ubiquitously but differentially expressed in various tissues (Fig. 2). The variations of OsFLZs suggest that they may exert different functions in rice growth and development.

It has been reported that *AtFLZ* genes are transcriptionally regulated by multiple biotic and abiotic stress treatments in *Arabidopsis* and maize (Jamsheer and Laxmi 2015; Jamsheer et al. 2018a; Chen et al. 2021), and plays important roles during plant growth or stress response (He and Gan 2004; Chen et al. 2013, 2021; Hou et al. 2013; Jamsheer et al. 2018a). However, the roles of *OsFLZ* genes in rice growth and development or their responses to environmental stresses are totally unknown. Studies in Arabidopsis showed that FLZs function as the scaffolding proteins of SnRK1 complex (Jamsheer et al. 2018b). Due to the central role of SnRK1 in the regulation of plant response to submergence stress (Lu et al. 2007; Lee et al. 2009; Cho et al. 2012; Chen et al. 2017), we reason that *OsFLZs* might play important roles in regulating seed germination and early seedling growth under submergence in rice. As predicted, our yeast two-hybrid assays showed that the selected eight representative OsFLZ proteins could strongly interact with SnRK1A (Fig. 4). Moreover, the eight *OsFLZ* genes are submergence-responsive (Fig. 5b), indicating the potential role of them in rice submergence response.

As a case study, we selected the most strongly submergence-responsive *OsFLZ* gene, *OsFLZ18*, for further functional studies. The phenotypic analysis results showed that the *OsFLZ18*-OE transgenic rice had shorter shoot in air and shorter coleoptiles under submergence conditions than those of the WT plants (Fig. 7), pointing out the critical role of *OsFLZ18* in rice growth and submergence response. The pull-down and BiFC data confirmed that OsFLZ18 interacted with SnRK1A in vitro and in vivo (Fig. 6a, b). Interestingly, the dual-luciferase assay showed that SnRK1A could significantly activate the expression of *αAmy3*, a positive regulator in rice flooding tolerance (Hwang et al. 1999), while the addition of OsFLZ18 could effectively antagonize the SnRK1A-dependent transcriptional activation of *αAmy3* (Fig. 6d). Consistent with this, the qRT-PCR results showed that the expression level of *αAmy3* was significantly decreased in *OsFLZ18-*OE lines (Fig. 7c). Based on these results, we conclude that OsFLZ18 negatively regulate SnRK1 activity to repress the expression of *αAmy3*, thus modulating rice seed germination and coleoptile elongation under submergence conditions.

In addition to the important role in submergence stress in rice, our current survey also found that many *OsFLZ* genes were differentially expressed when rice seedlings were subjected to desiccation, salinity and cold treatments (Additional file 3: Fig. S2), suggesting that they might also function in other abiotic stresses in rice. Thus, our current study provides fundamental information of OsFLZ proteins in rice. The results provide new insights in the mechanisms of rice seed germination and early seedling growth under low oxygen condition and can be a starting point for further functional characterizations of OsFLZ proteins in regulating rice responses to submergence and other abiotic stresses.

## Conclusions

In this study, our work identified 29 FLZ genes in rice, which can be divided into 4 clades. FLZ genes were differentially expressed in all tissues and most of them were submergence-responsive. Subcellular localization indicated that OsFLZ2, OsFLZ5, OsFLZ6, OsFLZ11, OsFLZ18, OsFLZ20 and OsFLZ27 were localized in both the nucleus and cytoplasm, while OsFLZ10 was shown to target the granular spots in the cytoplasm. Yeast two-hybrid experiment suggested eight typical OsFLZ proteins interact with SnRK1A, a central regulator of seed germination and seedling growth in rice. As a case study, phenotypic analysis results showed OsFLZ18 negatively regulate rice growth in both air and submergence conditions.

## Materials and Methods

### Data Search and Analyses

To obtain the sequencing data for all of the *OsFLZ* genes in rice, the PF04570.7 (DUF581) was queried in the Rice Genome Annotation Project website (RGAP) (http://rice.plantbiology.msu.edu/). Information about gene locus, chromosome location, nucleotide length, protein length, protein molecular weight, protein isoelectric point for each gene was also queried from the Rice Genome Annotation Project website. The protein sequences of *FLZ* family genes from *Arabidopsis* were downloaded from TAIR (https://www.arabidopsis.org/). A phylogenetic tree of OsFLZs and AtFLZs proteins based on amino acid sequences was constructed using the Neighbor-Joining algorithm with 1000 bootstrap replicates in MEGA 7.0. Multiple sequence alignments of the FLZ domain of OsFLZs and AtFLZs were performed using MEGA 7.0 and reordered according to the results of phylogenetic analysis by manual adjustment using GeneDoc.

### RNA Extraction and Quantitative Real-Time (qRT-PCR) Assay

For qRT-PCR analysis, embryos of *Nipponbare* were collected from dry seeds and up to 2 d after germination, after which only the primary coleoptiles were collected (4 d to 8 d samples). Each sample including 15 plants was used for RNA extraction. Total RNA was extracted from the collected samples using the Hipure plant RNA Mini Kit (Magen), according to the manufacturer’s instructions. First-strand cDNA was synthesized using the PrimeScriptTM RT Reagent Kit with gDNA Eraser (TaKaRa). Ten times-diluted cDNA samples were used for qRT-PCR amplification in 10 μL reaction volumes with SYBR Green PCR Master Mix (TB Green Premix Ex Taq II, Takara). Quantitative real-time PCR was run on the CFX Connect system (Bio-RAD) using the following reaction conditions: 95 °C for 1 min followed by 50 cycles of 95 °C for 10 s and 60 °C for 15 s. To normalize the variance among samples, the rice *OsEF1α* gene was used as the internal control, and relative expression levels of genes were calculated using the 2^−△△CT^ method (Livak and Schmittgen 2001). The primers used in this experiment are listed in Additional file 4: Table S2.

### Subcellular Localization Assay

Firstly, the subcellular localizations of OsFLZs were predicted using the WoLF PSORT web server (http://www.genscript.com/psort/wolf_psort.html). To validate the subcellular localization, the full-length OsFLZs coding sequence was amplified and inserted into the pCAMBIA1300-Ubi-GFP, a binary vector containing a maize ubiquitin promoter sequence based on pCAMBIA1300, to generate an in-frame fusion between the *OsFLZs* and *GFP* genes. The *OsFLZs-GFP* constructs were introduced into *Agrobacterium tumefaciens* (strain GV3101). The culture of *Agrobacterium tumefaciens* containing relevant construct was infiltrated into leaf tissues of 4- to 5-week-old *Nicotiana benthamiana* plants with a 1-mL syringe. The transient expression of the OsFLZs-GFP fusion protein was observed using a Confocal Microscopy (LSM 810). The primers used to generate the corresponding constructs are listed in Additional file 4: Table S2.

### Yeast Two-Hybrid Assay

The yeast two-hybrid (Y2H) assay was performed following the manufacturer’s instructions (Clontech, http://www.clontech.com/). To generate the activation domain (AD)-fused OsFLZs, the full-length CDS of the respective genes were fused in-frame to pGADT7 vector and confirmed via sequencing. For DNA-binding-domain (BD)-fused SnRK1A, the full-length CDS of *SnRK1A* were amplified and cloned into pGBKT7 vector and confirmed via sequencing. Yeast AH109 cells were co-transformed with each set of different vector combinations as indicated. The plasmid combination of pGBKT7-53 and pGADT7-T was used as a positive control. All yeast transformants were grown on –Trp/–Leu medium and screened on SD/-Trp/-Leu/-His with 1 mM 3-amino-1,2,4-triazole (3-AT) medium or SD/-Trp/-Leu/-His/-Ade medium for interaction test. The primers used in this study are listed in Additional file 4: Table S2.

### Pull-Down Assay

For in vitro pull-down assays, the pGEX4T-3-OsFLZ18 (GST-tagged OsFLZ18) and pRSETA-SUMO-SnRK1A (His-tagged SnRK1A) were constructed and expressed in the *Escherichia coli* BL21 (DE3) strain. Briefly, equal volumes of GST or GST-OsFLZ18 proteins were incubated with His- SnRK1A in pull-down buffer (50 mM Tris–HCl; 150 mM NaCl; 1 mM EDTA; 1% NP-40) at 4 °C for overnight, and then the GST-resin was washed for three times with pull-down buffer. The pulled proteins were eluted by boiling and further analyzed by immunoblotting using anti-GST and anti-His antibodies, respectively.

### Bimolecular Fluorescence Complementation (BiFC) Assay

For BiFC experiment, the full-length cDNA sequences of *OsFLZ18* and *SnRK1A* were cloned into the cYFP and nYFP vectors to generate OsFLZ18-cYFP and SnRK1A-nYFP constructs, respectively. The constructs were then transiently expressed in 4- to 5-week-old *Nicotiana benthamiana* cells using *Agrobacterium strain* GV3101. After incubation for 72 h, the YFP fluorescence was observed using a Confocal Microscopy (LSM 710). The primers used in this study are listed in Additional file 4: Table S2.

### Dual-Luciferase Reporter Assay

For promoter-binding analysis, 2-kb *αAmy3* promoter regions up from the start code ATG were cloned into pGreenII 0800-LUC to drive the *LUC* gene. The full-length coding regions of OsFLZ18 and SnRK1A were inserted into pGreenII 62-SK to generate the effector constructs, respectively. The effector and reporter constructs were co-transfected into Arabidopsis protoplast cells, and relative transcriptional activity of the promoters of *αAmy3* were calculated as the ratio of *LUC* to *REN* using the Dual-Luciferase Reporter Assay System (Promega) in accordance with the manual.

### Plant Materials and Growth Conditions

To overexpress *OsFLZ18* in rice, the pCAMBIA1300-Ubi-*OsFLZ18-GFP* vector was introduced into *Nipponbare* by *Agrobacterium*-mediated genetic transformation and the T_3_ homologous lines were used for phenotypic analysis as following: The healthy and filled seeds were incubated at 49 °C for 96 h to break dormancy. Rice seeds were sterilized with diluted bleach (20-min incubation in 2.5% sodium hypochlorite, rinsing and washing in sterile water 3 times). Afterwards, seeds were germinated at 28 °C in the dark conditions. For germination in air, seeds were germinated in capped glass tubes. For germination under submergence, seeds were placed in glass tubes, 10 cm of autoclaved water was carefully poured into the tube, and then the tubes were sealed with lids. After 7 days, the lengths of coleoptiles were measured and the seedlings were photographed. These experiments were conducted with three independent biological replicates.

## Supplementary Information

**Additional file 1: Table S1.** Overview of *OsFLZ* genes identified in rice.

**Additional file 2: Figure S1.** Chromosomal distribution of *OsFLZ* genes in rice. The chromosomal positions of the *OsFLZ* genes are indicated by their generic names. Black ovals on the chromosomes indicate the rough position of centromeres. Chromosome numbers are showed on the top of each chromosome. The ruler on the left indicates the physical map distance among genes (Mb).

**Additional file 3: Figure S2.** Heatmap of the gene expression of *OsFLZs* upon desiccation, salt and cold treatment in rice seedlings. The raw data of gene expression were downloaded from The Bio-Analytic Resource for Plant Biology (http://bar.utoronto.ca/).

**Additional file 4: Table S2.** Primers used in this study.

## Data Availability

The datasets supporting the conclusions of this article are provided within the article and its additional files.

## Abbreviations

*αAmy3*: Alpha-amylase gene 3
BiFC: Bimolecular fluorescence complementation
Chl: Chloroplast
FLZ: FCS-like zinc finger proteins
GFP: Green fluorescent protein
QTL: Quantitative trait loci
SnRK1A: Sucrose nonfermenting1-related protein kinase 1a
Y2H: Yeast two hybrid
