# Supplementary material for: Genome-Wide Identification, Expression and Functional Analysis Reveal the Involvement of FCS-Like Zinc Finger Gene Family in Submergence Response in Rice
Source: Rice (N Y). 2021 Aug 21;14:76. doi: 10.1186/s12284-021-00519-3 (PMC8380221; doi:10.1186/s12284-021-00519-3)
Supplement: Supplementary file 1 — Additional file 1: Table S1. Overview of OsFLZ genes identified in rice. [file 12284_2021_519_MOESM1_ESM.pdf]

Table S1 Overview of *OsFLZ* genes identified in rice

Gene ID		DNA attributes		Protein attributes			
Gene name[a]	Gene locus (MSU)[b]	Chromosome Location[c]	Nucleotide length (bp)	Length (aa)	MW (kDa)	PI	Subcellular localization[d]
OsFLZ1	LOC_Os01g08520	Chr1: 4217547 - 4215032	1167	389	39.42	5.28	Chlo, ER
OsFLZ2	LOC_Os01g41010	Chr1: 23224351- 23226620	783	261	28.60	4.07	Nucl, Chlo, Extr
OsFLZ3	LOC_Os01g52100	Chr1: 29960694 - 29963067	789	263	28.34	4.41	Nucl, Chlo, Cyto
OsFLZ4	LOC_Os02g07820	Chr2: 4095630 - 4098081	657	219	23.02	5.32	Nucl
OsFLZ5	LOC_Os02g37970	Chr2: 22943822 - 22942416	381	127	13.72	8.62	Chlo, Nucl, Mito
OsFLZ6	LOC_Os02g46180	Chr2: 28137459 - 28136329	441	147	16.09	8.51	Nucl, Chlo
OsFLZ7	LOC_Os02g46190	Chr2: 28144661 - 28144188	378	126	14.29	7.45	Chlo, Mito, Nucl
OsFLZ8	LOC_Os02g46210	Chr2: 28154641 - 28153272	321	107	11.49	5.12	Chlo, Nucl, Cyto
OsFLZ9	LOC_Os02g51550	Chr2: 31575808 - 31577576	444	148	15.16	8.83	Chlo, Mito, Nucl
OsFLZ10	LOC_Os03g08520	Chr3: 4380736 - 4379666	492	164	17.87	10.45	Nucl, Chlo, Mito
OsFLZ11	LOC_Os03g46260	Chr3: 26155504 - 26152125	909	303	31.78	6.85	Chlo, Mito
OsFLZ12	LOC_Os04g49620	Chr4: 29590515 - 29588213	453	151	15.70	5.98	Nucl, Chlo, Cyto
OsFLZ13	LOC_Os04g49650	Chr4: 29607165 - 29605672	360	120	13.63	9.81	Nucl, Chlo, Extr
OsFLZ14	LOC_Os04g49660	Chr4: 29611143 - 29610154	384	128	14.69	7.15	Nucl, Chlo, Mito
OsFLZ15	LOC_Os04g49670	Chr4: 29621500 - 29619361	423	141	15.88	6.69	Extr, Chlo, Cyto
OsFLZ16	LOC_Os04g49680	Chr4: 29625132 - 29624153	315	105	11.45	8.29	Chlo, Mito, Nucl
OsFLZ17	LOC_Os05g08800	Chr5: 4839861 - 4837556	984	328	34.01	5.77	Chlo, Mito
OsFLZ18	LOC_Os06g03520	Chr6: 1361364 - 1362509	435	145	15.47	7.37	Nucl, Chlo, Cyto
OsFLZ19	LOC_Os06g05970	Chr6: 2740452 - 2738395	612	204	21.97	6.34	Nucl
OsFLZ20	LOC_Os06g11980	Chr6: 6397221 - 6395388	429	143	15.03	9.12	Chlo, Nucl, Mito
OsFLZ21	LOC_Os06g14070	Chr6: 7845970 - 7845545	309	103	10.76	4.50	Nucl, Chlo, Mito
OsFLZ22	LOC_Os06g50080	Chr6: 30340446 - 30341829	411	137	14.63	7.39	Nucl, Mito, Chlo
OsFLZ23	LOC_Os07g42390	Chr7: 25364560 - 25363519	594	198	20.59	8.35	Chlo
OsFLZ24	LOC_Os08g31510	Chr8: 19491642 - 19490070	600	200	20.86	4.56	Chlo, Mito, Nucl
OsFLZ25	LOC_Os08g34984	Chr8: 22034104 - 22035394	576	192	20.60	11.56	Chlo, Cyto, Mito
OsFLZ26	LOC_Os09g20240	Chr9: 12126822 - 12128189	576	192	20.10	8.84	Nucl, Chlo
OsFLZ27	LOC_Os09g26370	Chr9: 15927025 - 15928164	525	175	19.61	10.23	Nucl, Mito, Cyto
OsFLZ28	LOC_Os10g28680	Chr10: 14962880 - 14960578	903	301	31.79	5.22	Nucl, Mito, Chlo:
OsFLZ29	LOC_Os11g43790	Chr11: 26453036 - 26453881	453	151	15.88	8.59	Chlo, Mito

[a] Rice FLZs were designated as *OsFLZ1-OsFLZ29*

[b] Accession number of RGAP (http://rice.plantbiology.msu.edu/) locus ID

[c] Position of gene in the genomic fragment

[d] Subcellular localization of OsFLZs supported by WoLF PSORT (http://www.genscript.com/psort/wolf_psort.html)

Nucl, nuclear; Cyto, cytoplasm; Chlo, chloroplast; Mito, mitochondrion; Extr, extracellular; ER, endoplasmic reticulum
