# Supplementary figures and images for: Genome-Wide Identification, Expression and Functional Analysis Reveal the Involvement of FCS-Like Zinc Finger Gene Family in Submergence Response in Rice
Source: Rice (N Y). 2021 Aug 21;14:76. doi: 10.1186/s12284-021-00519-3 (PMC8380221; doi:10.1186/s12284-021-00519-3)

Figure S1

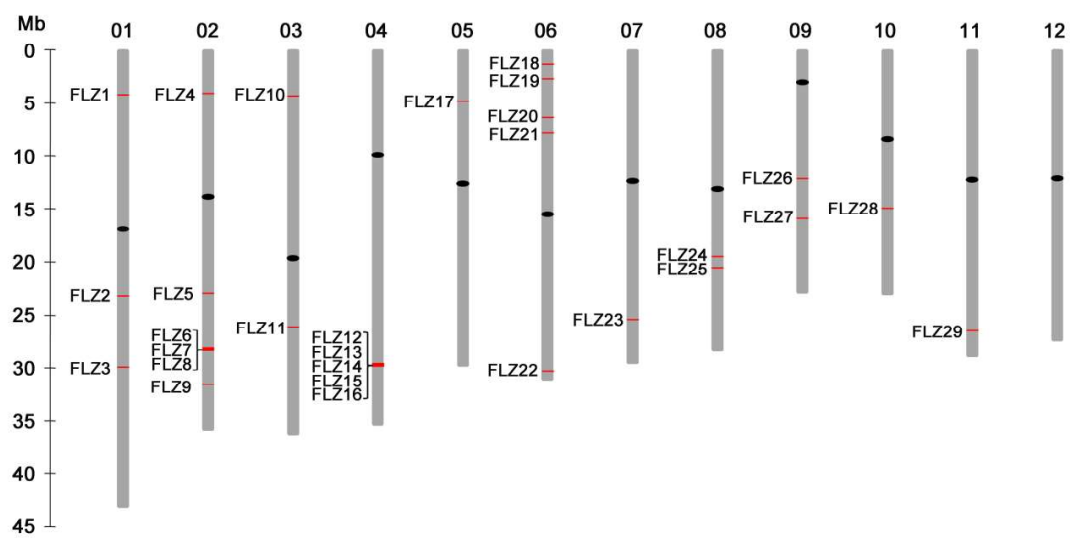

Supplement: Supplementary file 2 — Additional file 2: Figure S1. Chromosomal distribution of OsFLZ genes in rice. The chromosomal positions of the OsFLZ genes are indicated by their generic names. Black ovals on the chromosomes indicate the rough position of centromeres. Chromosome numbers are showed on the top of each chromosome. The ruler on the left indicates the physical map distance among genes (Mb). [file 12284_2021_519_MOESM2_ESM.pdf]

Figure S2

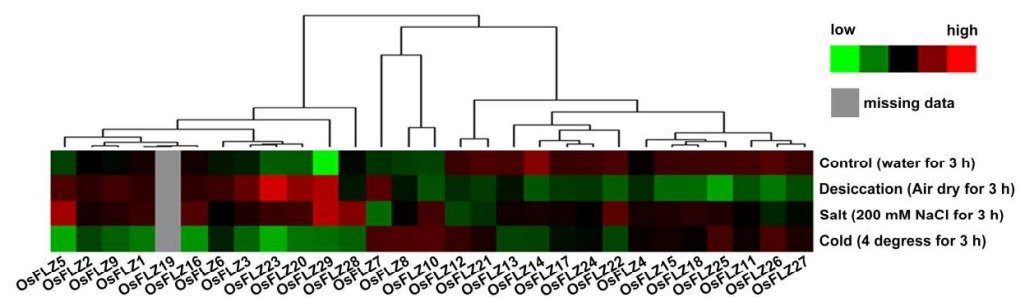

Supplement: Supplementary file 3 — Additional file 3: Figure S2. Heatmap of the gene expression of OsFLZs upon desiccation, salt and cold treatment in rice seedlings. The raw data of gene expression were downloaded from The Bio-Analytic Resource for Plant Biology (http://bar.utoronto.ca/). [file 12284_2021_519_MOESM3_ESM.pdf]
