# Supplementary material for: Genome-Wide Identification, Expression and Functional Analysis Reveal the Involvement of FCS-Like Zinc Finger Gene Family in Submergence Response in Rice
Source: Rice (N Y). 2021 Aug 21;14:76. doi: 10.1186/s12284-021-00519-3 (PMC8380221; doi:10.1186/s12284-021-00519-3)
Supplement: Supplementary file 4 — Additional file 4: Table S2. Primers used in this study. [file 12284_2021_519_MOESM4_ESM.pdf]

Table S2

The primers used in this study.

Primer name	Sequence
OsFLZ2-Y2H-F	CAGTGAATTCCACCCG ATGGCAGCTGAATCCTCCCT
OsFLZ2-Y2H-R	TATCGATGCCCACCC TCACTGGATCAGTTGGAAAAT
OsFLZ5-Y2H-F	CAGTGAATTCCACCCG ATGGCCGGCCTGAGTGTCCTT
OsFLZ5-Y2H-R	TATCGATGCCCACCC TCAGTACGCGAAGCCACCGGCG
OsFLZ6-Y2H-F	CAGTGAATTCCACCCG ATGGACTTCACCTCGTCCTACTTCC
OsFLZ6-Y2H-R	TATCGATGCCCACCC CTACCTCTGCGGCTGCAGCT
OsFLZ10-Y2H-F	CAGTGAATTCCACCCG ATGATTCCGAGAGCTCCGAG
OsFLZ10-Y2H-R	TATCGATGCCCACCC TCATGAAGCAGCGATCTGCAG
OsFLZ11-Y2H-F	CAGTGAATTCCACCCG ATGCTTCGCAACAGATCGAG
OsFLZ11-Y2H-R	TATCGATGCCCACCC CTAGCGCAAATTGTCCACCGC
OsFLZ18-Y2H-F	CAGTGAATTCCACCCG ATGATGGAGTCGAGGTACGTCA
OsFLZ18-Y2H-R	TATCGATGCCCACCC TCACACAGTGCCAGACACGG
OsFLZ20-Y2H-F	CAGTGAATTCCACCCG ATGACGACGCTGGGGAAGAGGCAC
OsFLZ20-Y2H-R	TATCGATGCCCACCC CTAGGCGGCGGCTACGGCGC
OsFLZ27-Y2H-F	CAGTGAATTCCACCCG ATGCTTCCGAGCCGGCAGAGA
OsFLZ27-Y2H-R	TATCGATGCCCACCC CTAGGCTACGGCGAGTAGTCTCC
SnRK1A-BD-F	CAGTGAATTCCACCCG ATGGAGGGAGCTGGCAGAGAT
SnRK1A-BD-R	TATCGATGCCCACCC TTAAAGGACTCTCAGCTGAGT
OsFLZ27-GFP-F	GATTAACAGGGATCCCCC ATGCTTCCGAGCCGGCAGAGA
OsFLZ27-GFP-R	GAGACTAGTGGTACCCCC GGCTACGGCGAGTAGTCTCC
OsFLZ20-GFP-F	GATTAACAGGGATCCCCC ATGACGACGCTGGGGAAGAG
OsFLZ20-GFP-R	GAGACTAGTGGTACCCCC GGCGGCGGCTACGGCGCCGC
OsFLZ18-GFP-F	GATTAACAGGGATCCCCC ATGATGGAGTCGAGGTACGTCAAG
OsFLZ18-GFP-R	GAGACTAGTGGTACCCCC CACAGTGCCAGACACGGCGG
OsFLZ11-GFP-F	GATTAACAGGGATCCCCC ATGCTTCGCAACAGATCGAG
OsFLZ11-GFP-R	GAGACTAGTGGTACCCCC GCGCAAATTGTCCACCGCCT
OsFLZ10-GFP-F	GATTAACAGGGATCCCCC ATGATTCCGAGAGCTCCGAG
OsFLZ10-GFP-R	GAGACTAGTGGTACCCCC TGAAGCAGCGATCTGCAGCCT
OsFLZ6-GFP-F	GATTAACAGGGATCCCCC ATGGACTTCACCTCGTCCTACTTC
OsFLZ6-GFP-R	GAGACTAGTGGTACCCCC CCTCTGCGGCTGCAGCTGAC
OsFLZ5-GFP-F	GATTAACAGGGATCCCCC ATGGCCGGCCTGAGTGTCCTT
OsFLZ5-GFP-R	GAGACTAGTGGTACCCCC GTACGCGAAGCCACCGGCGC
OsFLZ2-GFP-F	GATTAACAGGGATCCCCC ATGGCAGCTGAATCCTCCCT
OsFLZ2-GFP-R	GAGACTAGTGGTACCCCC CTGGATCAGTTGGAAAATAC
OsFLZ27-qPCR-F	ATGCTTCCGAGCCGGCAGAGAA
OsFLZ27-qPCR-R	ATACCGTGATCGCCATGGCC
OsFLZ20-qPCR-F	ATGACGACGCTGGGGAAGAG
OsFLZ20-qPCR-R	CCTGCTGCTCCTCCTCCTCC
OsFLZ18-qPCR-F	ATGATGGAGTCGAGGTACGT
OsFLZ18-qPCR-R	AGGTAGTGGTAATCGCCGTC

OsFLZ11-qPCR-F	CGCAACAGATCGAGGAGATC
OsFLZ11-qPCR-R	AAGAGGAGGAAGACGACGAC
OsFLZ10-qPCR-F	ATGATTCCGAGAGCTCCGAG
OsFLZ10-qPCR-R	TGTATGATCAGCCGGAGCCC
OsFLZ6-qPCR-F	CCTACTTCCACGCCTTCGGCAA
OsFLZ6-qPCR-R	AACGTTCACCGCCTTCGCGC
OsFLZ5-qPCR-F	CCGGCCTGAGTGTCCTTCTT
OsFLZ5-qPCR-R	GAGGACATCGTCTCTGGGTG
OsFLZ2-qPCR-F	ATGGCAGCTGAATCCTCCCT
OsFLZ2-qPCR-R	GCATCCAGCTCAATCAAACA
SnRK1A- qPCR-F	GAGACACCAAACTCAGCCACTG
SnRK1A-qPCR-R	ATGCCTCAAGCCAAACCCAG
αAMY3-qPCR-F	GCTCAAACCAGTTTCTACACGGC
αAMY3-qPCR-R	GCCCCGCAATTAACCTAGAGG
SnRK1A-mcherry-F	GACGAGCTGTACAAGACTAGT ATGGAGGGAGCTGGCAGAGA
SnRK1A-mcherry-R	GAGCTCGCTCTCGAGGGTACC TTAAAGGACTCTCAGCTGAG
FLZ18-GST-F	ATCTGGTTCCGCGTGGATCCATGATGGAGTCGAGGTAC
FLZ18-GST-R	ACCCGGGAATTCGGGGATCCTCACACAGTGCCAGACAC
SnRK1A-his-sumo-F	CAGGAACAAACCGGTGGATCC ATGGAGGGAGCTGGCAGA
SnRK1A-his-sumo-R	GCAGATGTCGAGCTCGGATCCTTAAAGGACTCTCAGCTG
SnRK1A-BiFC-F	CGACGGTACCGCGGGCCCATGGAGGGAGCTGGCAGA
SnRK1A-BiFC-R	GCTCACCATCAGGATCCCAAGGACTCTCAGCTGAGT
OsFLZ18-BiFC-F	CGACGGTACCGCGGGCCCATGATGGAGTCGAGGTAC
OsFLZ18-BiFC-R	CACGCTGCCCAGGATCCCCACAGTGCCAGACACGGC
αAMY3-F	ATCGAATTCCTGCAGCCCtaccttttctctttctcagg
αAMY3-R	AGAACTAGTGGATCCCCCatatagaacaccgggggcac
EF1α-F	TTTCACTCTTGGTGTGAAGCAGAT
EF1α-R	GACTTCCTTCACGATTTCATCGTAA
